# Advancements and challenges in the management of chronic inducible urticaria

**DOI:** 10.3389/fimmu.2026.1747542

**Published:** 2026-04-24

**Authors:** Menghao Zhang, Juan Liu, Yuxiang Zhi

**Affiliations:** Department of Allergy, Peking Union Medical College Hospital, Chinese Academy of Medical Sciences and Peking Union Medical College, Beijing, China

**Keywords:** anti-KIT biologics, biomarkers, Bruton’s tyrosine kinase inhibitor, chronic inducible urticaria, disease-specific assessment tools

## Abstract

Chronic inducible urticaria (CIndU) is a group of diseases characterized by recurrent wheals and/or angioedema induced by specific triggers. Research into its underlying mechanisms remains underdeveloped, historically lagging behind that of chronic spontaneous urticaria (CSU). The profound heterogeneity across CIndU subtypes presents a central challenge, translating into limited and variable efficacy of conventional therapies, including second-generation H1-antihistamines and omalizumab, highlighting the inadequacy of a uniform management strategy. Encouragingly, the clinical development of novel targeted agents, notably anti-KIT biologics and Bruton’s tyrosine kinase (BTK) inhibitors, now offers new promise. Concurrently, the refinement of disease-specific assessment tools and the exploration of biomarkers are facilitating a more personalized approach. This review summarizes recent advances and persistent challenges in CIndU management, advocating for a concerted effort to deepen mechanistic understanding, validate predictive biomarkers, and integrate patient stratification into future clinical trials, thereby paving the way for precision medicine in this field.

## Introduction

Chronic inducible urticaria (CIndU) is a group of chronic urticaria (CU) characterized by recurrent wheals and/or angioedema triggered by specific physical or non-physical stimuli (1), with an underlying pathological process mainly involving the hyperactivation activation of mast cells and bioactive mediator release (2, 3). CIndU has a prevalence of approximately 0.1~5% and accounting for 20-30% of CU cases (4–7). It frequently comorbid with chronic spontaneous urticaria (CSU) (2, 8) and CIndU patients may experience a natural course that lasts several years (e.g., the average cold urticaria (ColdU) duration has been reported to be approximately 6 years, and the disease may persist for 20 years or longer (9)), leading to prolonged illness and often associated psychiatric comorbidities such as anxiety and depression (10, 11), which further exacerbates the disease burden on the quality of life and social aspects of patients (1, 11–13), underscoring its important clinical status and the urgency for research. However, compared to CSU, the basic and clinical research on CIndU has long lagged.

CIndU encompasses a disease spectrum with high heterogeneity (14, 15). International classifications divide it into two major categories: physical and non-physical forms, with common subtypes including symptomatic dermographism (SD), cold contact urticaria (ColdU), and cholinergic urticaria (CholU) (1, 16). This heterogeneity is profoundly reflected in various aspects such as epidemiology, clinical manifestations, and treatment responses: overall, women have a higher prevalence (5, 17), but CholU is more common in men (16); clinically, mixed CIndU or patients with comorbid CIndU and CSU often have more complex conditions (8, 18), while the symptom spectrum and risk of systemic reactions differ substantially among different subtypes (e.g., patients with dermographism typically do not exhibit angioedema, while those with ColdU are more prone to systemic symptoms) (16). Although avoidance of triggers is the cornerstone of management, it often proves insufficient (19, 20). The treatment of CIndU remains a challenge. The second-generation H1-antihistamines (sgAHs) exhibits marked heterogeneity in efficacy (18, 21), with more than half of CIndU patients showing insufficient response to standard or even increased doses (12, 20, 22). Additionally, omalizumab is effective for some subtypes (23, 24), its application remains off-label, and efficacy also varies by subtype (25–27). This suggests that a “one-size-fits-all” clinical management strategy is inadequate to match the heterogeneous characteristics of CIndU. The root cause lies in our insufficient understanding of the unique pathogenesis mechanisms of CIndU subtype and the lack of biomarker to predict efficacy to guide decision-making.

Encouragingly, innovative therapies targeting key nodes of mast cell (MC) activation, such as KIT-targeting MC depletes (28–31) and Bruton’s tyrosine kinase (BTK) inhibitors (32, 33), are under clinical development and show great potential of CIndU therapy (12). Meanwhile, the optimization of disease-specific assessment tools (34–37) and exploration of predictive biomarkers (18, 38, 39) are paving the way for precise evaluation and treatment.

Therefore, this review aims to systematically integrate the latest advances in the pathogenesis, treatment strategies, and disease assessment of CIndU, with a particular emphasis on cross-subtype heterogeneity comparison. We will move beyond the traditional “one-size-fits-all” model and explore the potential for precision treatment based on intrinsic characteristics. We hope that by promoting in-depth investigations into unique mechanisms, validating predictive biomarkers, and advocating for the adoption of patient stratification strategies in clinical trial designs, we hope ultimately contribute to the diagnosis and treatment of CIndU into a new era of precision medicine.

## The pathogenesis of CIndU

The pathogenesis of CIndU is unclear, which appears to share similar pathogenic mechanisms that are involved in CSU (12). Strong evidence supports that MCs play a central role in the development of SD (28) and ColdU (40), and their mediators activate skin nerves and increase vascular permeability, which leads to pruritus, wheals, and/or angioedema. However, MCs can be activated via multiple receptor pathways, including c-KIT, Mas-related G protein–coupled receptor X2 (MRGPRX2), protease-activated receptors (PARs), sialic acid–binding immunoglobulin-like lectins (Siglecs), and cytokine receptors, any of which may contribute to the pathogenesis of CIndU (2). In addition, a study suggested that basophil activation was increasing in CIndU patients rather than healthy controls, which also emphasized the importance of basophils (41).

Specifically, the pathological mechanisms vary among the different subtypes of inducible urticaria. In SD, other potential pathological mechanisms beyond mast cell degranulation may be involved. These include alterations in coagulation parameters (such as prothrombin fragment F1 + 2, D-dimer, and soluble P-selectin), dysbiosis of the gut microbiota, and abnormalities in plasma lipid metabolism (42). While for CholU, the prevailing hypothesis implicates a complex interaction between several systems. This includes the activation of mast cells (releasing histamine), a hyper-response to cholinergic stimuli (such as acetylcholine), an autoimmune reaction to sweat components, and physical disruptions to sweating like poral occlusion or anhidrosis, all of which are believed to contribute to the development of symptoms (43). SolU is driven by a photoallergic reaction: sunlight activates a chromophore, generating a photoallergen that triggers IgE-mediated mast cell degranulation, resulting in wheals and pruritus. Non-IgE pathways and genetic factors may also contribute (44). And DPU is driven by a mechanotransduction process where sustained skin pressure activates mechanosensitive ion channels (PIEZO1/PIEZO2) on mast cells. This is enhanced by IL-33, leading to Ca^2+^-dependent mast cell degranulation and release of histamine, leukotrienes, and cytokines. The condition resembles a late-phase reaction (LPR): 4–12 hours post-stimulus, there is persistent mast cell activation with infiltration of eosinophils, neutrophils, and lymphocytes, causing deep dermal and subcutaneous inflammation (resulting in deep swellings). Endothelial cells upregulate adhesion molecules (e.g., ICAM-1, VCAM-1), promoting inflammatory cell recruitment. Multiple mediators (e.g., TNF-α, IL-5, IL-17, leukotrienes) are elevated, while others (IL-8, IL-13) are reduced (45).

Furthermore, the autoallergy hypothesis posits that endogenous autoallergens, by cross-linking IgE on MCs, may contribute to the pathogenesis of SD, ColdU, CholU, and SolU through a mechanism resembling type I hypersensitivity (44, 46). In ColdU, autoantibodies may act as cryoglobulins, which activate MCs upon cold exposure. This activation may occur through several mechanisms: immune complexes can induce MC degranulation either via complement activation (generating C5a that binds to the C5a receptor) or by cross-linking activating Fcγ receptors (FcγR). Besides, it is hypothesized that the cold-sensing TRP cation channels, transient receptor potential melastatin 8 (TRPM8) and transient receptor potential ankyrin 1 (TRPA1), may serve as the initial trigger for mast cell degranulation in ColdU (40). Additionally, unbound anti-IgE antibodies may cross-link IgE bound to the high-affinity IgE receptor (FcϵRI) (47, 48).

Beyond MCs and basophils, as mentioned before, a mixture of eosinophils, neutrophils, lymphocytes, and along with their related cytokines, such as IL-4, IL-5, and IL-33, was also shown in the skin biopsy samples from CU patients (49).

Altogether, the pathogenesis of CIndU involves a complex network in which specific triggers activate skin MCs via multiple receptor pathways, leading to the release of histamine and other inflammatory mediators. A deeper understanding of subtype-specific differences within this network is essential to advance CIndU toward a precision medicine framework.

## Current challenges of CIndU management

The overarching management principle for CIndU involves trigger avoidance and active symptomatic control (27). However, current therapeutic approaches face several challenges (20, 27). First-line treatment with sgAHs exhibits marked efficacy heterogeneity, with over half of patients responding inadequately to standard or even increased doses (18, 50). Although omalizumab is frequently used after antihistamine failure, its application in patients with isolated CIndU remains off-label, and its effectiveness varies considerably across subtypes (39, 51–53). Additionally, immunosuppressive therapies are limited by a scarcity of high-level evidence and safety concerns. Therefore, establishing novel, individualized treatment strategies tailored to subtype characteristics represents a central challenge in CIndU management.

## Antihistamine

In clinical practice, sgAHs are generally regarded as the initial pharmacologic intervention for CIndU (27, 54, 55). However, their efficacy is substantially limited by suboptimal overall response rates and considerable variability across subtypes (20) (**Table 1**). Robust clinical evidence indicates that more than 50% of CIndU patients do not respond adequately to standard-dose sgAHs (18, 50, 56).

**Table 1:** Antihistamine therapy in different CIndU.

Study	Antihistamine	Type of CIndU	Dosage	Number of subjects	effectiveness
Kocatürk E et al, 2017	Desloratadine Levocetirizine Rupatadine Hydroxyzine Cetirizine Ebastine Fexofenadine Loratadine Pheniramine maleate	SD, CholU, ColdU, Aquagenic urticaria	standard doses	70 CIndU vs. 66 CSU	20.9% vs. 37.9%
Ornek Ozdemir S et al, 2024	Unspecified	SD, ColdU, CholU	standard doses	296 CIndU vs. 127 CSU + CIndU	83.2% vs. 78.3 vs. 60.9% (SD vs. ColdU vs. CholU)
Gastaminza G et al, 2019	cetirizine	CholU	20 mg	22 CholU	unresponsive
Ghazanfar MN et al, 2020	Unspecified	CholU	up dosed (unspecified)	19 CholU vs. 8 CSU + CholU vs. 9 CholU + CIndU	unresponsive
Mellerowicz E et al, 2019	Cetirizine, Loratadine, Desloratadine, Fexofenadine, Levocetirizine, Rupatadine, Ebastine	CholU	standard doses	111 CholU	Only 27.7% responsive
Miles LM et al, 2021	Unspecified	ColdU, CholU, SU, DPU	standard doses (72%) double doses (16%) four-doses (3%)	40 ColdU + 27 CholU + 10 SolU + 2 DPU + 10 (ColdU + CholU)	71.4% responsive
Kulthanan K et al, 2020	Cetirizine, desloratadine	DPU	Cetirizine 10 mg thrice daily, Desloratadine 5 mg/d	14 in Cetirizine, 11 in Desloratadine	Cetirizine 10 mg thrice daily was more effective than placebo, 3 of 11 patients response to Desloratadine 5 mg/d
Abajian M et al, 2016	Rupatadine	ColdU	20 mg/day vs. 40 mg/day	23	Both effective
Qian T et al, 2022	Ebastine	ColdU, CholU	10, 20, 40 mg/day	1 ColdU + 8 CholU	75% responsive to 10mg in CholU
Imamura S et al, 2024	Unspecified	SU	Unspecified	29	75.8% response to H1 antihistamine
Krause K et al, 2013	Bilastine	ColdU	20, 40, 80 mg/day	20	95% responsive

SD, Symptomatic Dermographism; CSU, Chronic Spontaneous Urticaria; CholU, Cholinergic Urticaria; ColdU, Cold Urticaria; CIndU, Chronic Inducible Urticaria; SU, Solar Urticaria; DPU, Delay Pressure Urticaria.

A multicenter prospective study comparing CIndU and CSU patients revealed a significantly lower response rate to standard-dose-sgAHs in the CIndU patients (20.9% vs. 37.9%) (50). Moreover, therapeutic responses exhibit pronounced subtype-specific heterogeneity (18). For instance, SD demonstrates relatively favorable response rates (18, 54), whereas CholU (57, 58) and delayed pressure urticaria (DPU) show lower efficacy (18, 18, 59–61). A meta-analysis of 21 studies involving 376 patients further highlighted these limitations, reporting a complete response rate of only 7.6% in SolU patients treated with sgAHs (62), the challenge of achieving full disease control with antihistamine monotherapy in many cases. This differential efficacy likely reflects the distinct, often non-histamine-driven, pathogenic mechanisms underlying various CIndU subtypes (15, 63–65).

For CU patients with an inadequate response to standard doses, current guidelines recommend increasing sgAHs up to four-fold in CU (1, 1, 6, 19, 66–68). A prospective controlled study indicates that patients with both CIndU and CSU showed similar responses to combining or updosing sgAHs (50). Evidence from ColdU and CholU also suggests that updosing sgAH might be effective (26, 60, 67, 69–72). Nevertheless, there are also some CIndU patients remain resistant even to fourfold doses sgAHs (58, 60, 73), and this proportion will rise when angioedema is present (6). Concomitant CSU and CIndU has been associated with antihistamine resistance, requiring more frequent treatment after 5 years or higher doses of sg-AH (55, 56). Despite these findings, the field still lacks individualized, subtype-specific dose-adjustment guidelines. Therefore, determining the optimal therapeutic dosage based on clinical subtype remains a pivotal challenge for optimizing the effectiveness of conventional antihistamine therapy.

## Anti-IgE therapy

Beyond sgAHs, no medications are currently licensed for isolated CIndU. Omalizumab, a monoclonal anti-IgE antibody, acts by reducing free IgE levels and downregulating the expression of the high-affinity IgE receptor (FcϵRI) on MCs and basophils, thereby inhibiting the release of inflammatory mediators (24). This mechanism is well-established, with substantial clinical evidence from 154–234 patients demonstrating its overall effectiveness in CIndU (24, 39, 53, 74), making it the most commonly used biologic for CIndU patients inadequately controlled by sgAHs (6, 26). However, its use remains off-label for patients with isolated CIndU (75), and its therapeutic benefits exhibit considerable heterogeneity across different subtypes (12, 27).

Meta analysis included 103 CIndU patients and clinical studies form multi-national centers enrolled in 234 patients have confirmed the safety of omalizumab, with an incidence of adverse events comparable to placebo (23, 53). A multinational, multicenter real-world study involving 234 CIndU patients showed that 73% of CIndU patients benefited from omalizumab, although complete response rates were generally lower than in CSU (39, 76). A slower onset of symptom improvement has also been noted in CIndU (39), with these patients (N = 29) representing a smaller proportion of early responders than CSU (4.3% vs. 95.7%) (77).

Randomized controlled studies have demonstrated rapid and robust effectiveness of omalizumab in patients with SD and ColdU: response time at 300 mg per 4 weeks was fast in 57% (N = 80) of complete/satisfactory responders (52). Additionally, retrospective studies have documented its effectiveness in certain patients with SolU (N = 30), DPU (N = 322), and CholU (N = 31, 3 centers) (58, 59, 78–80). In contrast, its efficacy in CholU and DPU is more limited and slower to manifest (57, 59). Research in CholU showed no significant short-term difference of omalizumab treatment from placebo, and an exercise challenge test negative rate of only 31.3% at week 48 (57), suggesting that these subtypes may involve more complex, non-IgE-driven pathogenic mechanisms. Currently, there are only a few case reports evaluating the efficacy of omalizumab 300 mg every 4 weeks for the treatment of antihistamine-refractory aquagenic urticaria (81) and 450 mg biweekly for heat urticaria (82), robust evidence of efficacy is lacking.

Currently, there are no reliable predictors for omalizumab response in CIndU. Optimizing omalizumab therapy for CIndU requires adjusting doses based on subtype and individual response, aiming for complete symptom control before reducing the dose or extending intervals (6). For patients not responding well to the standard 300 mg every 4 weeks, especially those with subtypes like CholU and DPU, increasing the dose to 450 mg or 600 mg or shortening the interval has improved symptom control in real-world studies (52, 62, 83, 84). A Chinese expert consensus (6) suggests omalizumab doses of 150–600 mg every 4 weeks for common CIndU subtypes (ColdU, CholU, SD) and at least 150 mg for SolU, adjusted based on clinical response. For subtypes with limited evidence, like heat-induced urticaria, dosing should be based on individual assessment. Current literature consists primarily of case reports indicating that patients with heat-induced urticaria have experienced significant clinical improvement following the administration of 300–450 mg of omalizumab every 2–4 weeks (82, 85). Retreatment with omalizumab can quickly and significantly improve symptoms in patients with CIndU who relapse after stopping treatment (86). Additionally, a study showed that extending omalizumab treatment to 24 weeks improved response rates in CIndU patients unresponsive at 12 weeks (50, 87), highlighting the need for sufficient treatment duration before deeming it ineffective.

## Alternative treatment

In addition to omalizumab and sgAHs, some alternative treatment options show certain potential in specific types of CIndU (26, 88, 89), but the evidence from evidence-based medicine is generally weak, necessitating extreme caution in clinical application.

Systemic immunomodulatory treatments, including cyclosporine, are primarily informed by research on CSU and expert clinical experience, yet there is a notable absence of guidelines explicitly endorsing their application in CIndU (89, 90). The evidence supporting their efficacy is predominantly derived from small-scale reports or case studies, lacking systematic validation through large-scale randomized controlled trials. While systemic corticosteroids can alleviate symptoms such as DPU, their prolonged use is constrained by a significant range of adverse effects (26). Additionally, agents such as colchicine, dapsone, sulfasalazine, montelukast, intravenous immunoglobulin, methotrexate, tripterygium glycosides, TNF inhibitors, and certain antibiotics are employed off-label in refractory CIndU cases (91–98). CU refractory to both standard and high-dose antihistamines remain a therapeutic challenge. CU refractory to both standard and high-dose antihistamines remain a therapeutic challenge. Current guidelines offer differing perspectives on the management of such patients. The EAACI guidelines do not recommend combining non-sedating antihistamines (nsAHs), citing insufficient evidence regarding the efficacy and safety of such regimens (99). In contrast, the American practice parameters recommend either up dosing of sgAHs or the addition of another sgAH, an H2-antihistamine, a leukotriene receptor antagonist (LTRA), or a first-generation H1-antihistamine (100). Clinical evidence supporting combination strategies, however, remains limited in certain respects. Although the use of 4-fold sgAHs in combination with H2-antihistamines, LTRAs, or both has been documented in patients with CSU, the optimal duration of such treatment has not been specified (56, 68, 101). Evidence for combination therapy in CIndU is even more limited, consisting primarily of sporadic case reports. For instance, symptom control in DPU was achieved with a triple regimen comprising a second-generation antihistamine, a leukotriene receptor antagonist, and cyclosporine (102). In SolU, combination regimens of antihistamines plus a LTRA, with or without omalizumab, have been reported to be effective (62, 80). Whereas combination therapy is rarely reported in CholU and ColdU, the available evidence collectively suggests a parallel to refractory CSU: the mainstay of combination treatment for refractory CIndU also involves antihistamines plus an LTRA or omalizumab. However, these substances are supported by very low levels of evidence, and their mechanisms of action in symptom amelioration remain unclear, necessitating confirmatory comparative studies. A multicenter phase II study has even demonstrated that a single course of intravenous immunoglobulin (2g/kg) is inadequate for sustained disease control in SolU (N = 9) (103). Furthermore, prolonged or immunosuppressive therapy can lead to significant adverse reactions in CU (7, 90). Ciclosporin, for example, can cause renal toxicity and hypertension in CU (104). Consequently, CU guidelines stress the importance of balancing efficacy with potential side effects and closely monitoring patients during systemic immunomodulatory therapy (1).

Conversely, desensitization therapies targeting specific trigger factors have been historically investigated, such as inducing tolerance to ColdU via cold baths or managing SolU through repeated and progressively increased UV exposure (88, 105, 106). In recent years, a limited number of studies have indicated that repeated induction of sweating may be beneficial for certain patients with CholU attributed to sweat allergy (15). However, this strategy poses a potential risk of anaphylaxis during the desensitization process and generally necessitates long-term maintenance treatment to solidify tolerance (62, 107), which raises concerns regarding their safety and applicability. Additionally, there have also been reports of systemic adverse reactions, such as dizziness and laryngeal edema, following high-dose UVA exposure (62). As a consequence, these strategies are infrequently used in clinical settings today, with treatment strategies now prioritizing the importance of identifying and avoiding known triggers.

In general, while these alternative options, which lack substantial evidence, offer more choices for some difficult-to-treat patients, their use should be carefully considered in terms of potential benefits and risks and should be closely monitored.

## Emerging targeted therapies in CIndU

**Figure 1:**
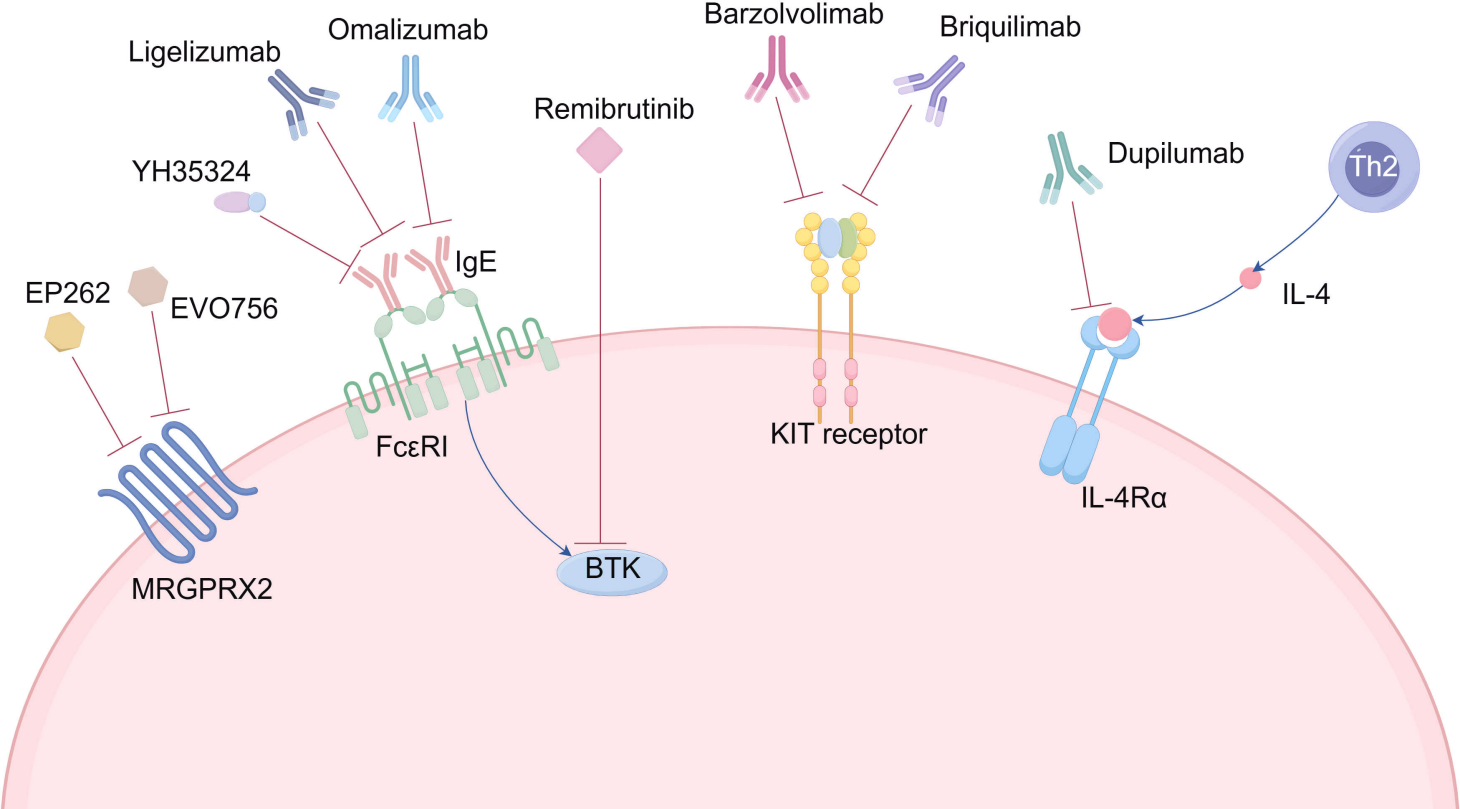
Available and novel targeted therapies under development for CIndU.

The clinical development of emerging targeted therapies for CIndU is advancing beyond traditional methods (**Figure 1**). Given the scarcity of studies focusing exclusively on CIndU, the conceptual framework of this figure was informed by recent reviews on targeted therapies in CSU (104, 108, 109), as mechanistic overlap exists between the two conditions and mixed CU studies occasionally include CIndU patients. We only show therapeutic agents supported by evidence from studies that explicitly enrolled CIndU patients—whether from CIndU-focused trials or mixed CU studies with clearly reported CIndU populations. Agents supported solely by CSU evidence were excluded. For example, Ligelizumab, a next-generation high-affinity humanized monoclonal anti-IgE antibody, has been reported to successfully treat patients with refractory and disabling SolU (110). Furthermore, the IgE-Trap protein (YH35324), which exhibits high affinity for free serum IgE, is currently undergoing evaluation in a Phase I trial involving patients with ColdU (NCT05960708) (12), offering new hope for CIndU treatment.

Additionally, promising strategies in CIndU now encompass MC depletion via anti-KIT biologics (28–30), blocks the interleukin-4 receptor alpha (IL-4Rα) via dupilumab (111–114), inhibition of intracellular signaling through BTK blockers (32), and MRGPRX2 antagonism for IgE-independent pathways (**Table 2**). While the clinical translation of these approaches has encountered both successes and challenges, collectively they represent a significant shift towards precision medicine in the management of CIndU, with the aim of addressing the heterogeneity of treatment responses through mechanism-based interventions.

**Table 2:** Ongoing and completed interventional clinical trials of CIndU.

NCT number	Status	Phase	Population/cohort	Intervention	Primary endpoint	Enrollment (actual)	Study completion (actual/estimated)	Target/mechanism	Target class
NCT05976243	Active, not recruiting	3	Adults with SD, ColdU, or CholU, anti-H1 refractory	remibrutinib vs PBO	% pts with UCT≥12 at Wk12	362	Jun 2029	BTK inhibitor	Small molecule
NCT06865651	Recruiting	2	Adults with CU (CSU ± CIndU), anti-H1 refractory	remibrutinib vs PBO	% Δ in MC marker (tryptase) at Wk12	44	Oct 2027	BTK inhibitor	Small molecule
NCT05405660	Completed	2	Adults with SD or ColdU, anti-H1 refractory	barzolvolimab vs PBO	% pts with UAS7 = 0 at Wk12	196	Sep 2025	anti-KIT mAb	mAb
NCT06050928	Completed	1	Adults with SD or ColdU	EP262 vs PBO	% Δ in provocation threshold	33	Oct 2024	MRGPRX2 antagonist	Small molecule
NCT05960708	Completed	1	Pts with CSU or ColdU	YH35324; OMA; PBO	% pts with UAS7 = 0 at Wk12	30	Sep 2024	IgE-Trap	Fusion protein
NCT03749148	Completed	2	Pts with CholU	dupilumab vs PBO	% pts with UAS7 = 0 at Wk16	48	Feb 2023	IL-4Rα inhibitor	mAb
NCT04681729	Completed	3	Pts with ColdU, anti-H1 refractory	dupilumab vs PBO	Δ in Critical Temp Threshold at Wk12	82	Apr 2023	IL-4Rα inhibitor	mAb
NCT04853992	Completed	2	Pts with CholU	Izuforant vs PBO	Δ in induced-wheal count	20	Jul 2022	H4R antagonist	Small molecule

The data in this table is current as of 15 March 2026. Clinical trial information is sourced from ClinicalTrials.gov and the EU Clinical Trials Register. Final results for some recently completed studies may not yet have been published. pts, Patients; SD, Symptomatic Dermographism; CSU, Chronic Spontaneous Urticaria; CU, Chronic Urticaria; BTK, Bruton’s Tyrosine Kinase; CholU, Cholinergic Urticaria; ColdU, Cold Urticaria; CIndU, Chronic Inducible Urticaria; H4R, Histamine H4 Receptor; IgE, Immunoglobulin E; IL-4Rα, Interleukin-4 Receptor Alpha; IL-5Rα, Interleukin-5 Receptor Alpha; mAb, Monoclonal Antibody; MC, Mast Cell; MRGPRX2, Mas-related G protein-coupled receptor X2; OMA, Omalizumab; PBO, Placebo; UAS7, Urticaria Activity Score for 7 Days; UCT, Urticaria Control Test; Δ, Change from baseline; %, Percent; % pts, Proportion of patients.

## Targeting KIT for mast cell depletion

The recognition of the KIT signaling axis as a master regulator of MC biology has paved the way for a transformative therapeutic strategy in CIndU: direct depletion of the central effector cells (29, 115). This approach is spearheaded by barzolvolimab (28–30), a monoclonal antibody that targets the KIT receptor with high specificity (29). By potently inhibiting KIT signaling, barzolvolimab induces profound and sustained MC depletion (29), offering a novel mechanism of action beyond mediator blockade (30). It induces profound and durable MC depletion by high-affinity binding to c-KIT, thereby inhibiting stem cell factor (SCF)-mediated signaling.

Compelling clinical evidence for this strategy emerged from an open-label clinical trial (NCT04538794) (28). A remarkable 95% (19/20) of patients with ColdU or SD achieved a complete response following single intravenous infusion of barzolvolimab (3mg/kg, with a 12-week follow up). Barazolizumab was well tolerated except for changes in hair color and selective taste alterations. Of paramount clinical importance, this robust efficacy extended to CIndU patients who had previously demonstrated an inadequate response to omalizumab, highlighting its potential to address a significant unmet need in the treatment landscape. These promising findings have been further substantiated by the recently completed phase II study (NCT05405660) (31). At week 20, barzolvolimab demonstrated sustained and significant efficacy in patients with antihistamine-refractory CIndU (116). In patients with ColdU, complete response rates were achieved in 44.8% of those receiving 150 mg every 4 weeksand 67.7% of those receiving 300 mg every 8 weeks. Similarly, in patients with SD, complete response rates reached 51.6% and 53.6%, respectively (116). The favorable safety profile observed in this study, coupled with the robust efficacy data, has supported the advancement of barzolvolimab into phase III registrational studies, underscoring its potential to redefine the management landscape for CIndU by targeting the pathological core of mast cell-driven disease. The therapeutic arsenal targeting the KIT pathway continues to diversify. Briquilimab, another anti-KIT monoclonal antibody (117), is currently under investigation in an Ib/IIa trial (NCT06162728) for patients with CSU refractory or intolerant to omalizumab, potentially expanding the application of this drug class.

Collectively, these advancements underline the central role of skin MCs and KIT/SCF signaling in ColdU and SD and underscore the considerable promise of KIT-targeted therapies, which, through complementary mechanisms of cell depletion and signaling inhibition, are poised to redefine the management of CIndU by targeting its pathological core.

## Targeting the IL-4/IL-13 pathway

The exploration of IL-4Rα inhibition in CIndU presents a complex and nuanced clinical picture. Dupilumab, a monoclonal antibody that blocks the shared IL-4Rα subunit, thereby antagonizing the signaling of both IL-4 and IL-13, represents a compelling therapeutic strategy based on the established role of type 2 inflammation in various allergic diseases (118–121). Despite failing to meet the primary endpoint in a Phase III trial (LIBERTY-CSU CUPID B, NCT04180488) for patients with omalizumab-refractory CSU (118, 122), intriguing signals of efficacy have emerged from case reports in some refractory CIndU subtypes (113). For example, dupilumab has demonstrated effectiveness in isolated cases of ColdU (initial dose of 600 mg, followed by 300 mg alternate weekly) (111, 113) and CholU (600 mg as a loading dose and then 300 mg every 15 days) (114). Notably, a Phase II/III randomized controlled trial (NCT03749148) evaluating dupilumab specifically in CholU is ongoing. Conversely, a Phase III study in ColdU (NCT04681729) did not achieve its prespecified efficacy endpoints (114). The stark contrast between these outcomes underscores the profound heterogeneity within CIndU and highlights the critical necessity for future studies to refined patient stratification to identify the specific subpopulations most likely to benefit from this targeted approach.

## BTK inhibitors

BTK has emerged as a compelling therapeutic target in CU due to its pivotal role in FcϵRI signal transduction downstream of both IgE-dependent and IgE-independent activation pathways in MCs and basophils (123). Remibrutinib, a highly selective oral BTK inhibitor, has demonstrated rapid and substantial efficacy with 100mg bid in Phase IIb (NCT03926611) and 25mg twice daily in phase III (NCT05030311, NCT05032157) clinical trials for CSU (124–127). Importantly, a key mechanistic study provided compelling rationale for its potential broad application in CIndU, demonstrating that remibrutinib effectively inhibits the activation of human basophils and MCs stimulated by serum from both CSU and CIndU patients (32). Crucially, this inhibitory effect was independent of patients’ clinical response status to omalizumab, suggesting that BTK inhibition may be effective across a wider spectrum of CIndU subtypes, including those who refractory to anti-IgE therapy. While direct clinical evidence in CIndU populations is still being accumulated, this mechanistic promise, coupled with its established efficacy in CSU, positions BTK inhibitors as one of the most anticipated oral therapeutics in development for CIndU.

## MRGPRX2 antagonist

Beyond the targets discussed above, antagonists of the MRGPRX2, which mediates IgE-independent activation of MCs, basophils, and eosinophils (128), and MC degranulation (129), representing a promising new direction (130–132). The natural flavonoid fisetin has been shown to bind MRGPRX2 and inhibit MC activation (130), providing preclinical proof-of-concept for this approach in CU. Currently, MRGPRX2 antagonist EVO756 has completed a Phase IIa study (NCT06603220) in CIndU, including SD and ColdU. Concurrently, a Phase Ib study (NCT06050928) is underway evaluating another antagonist EP262 in the same CIndU subtypes. Positive findings from the Phase II trial of EVO756 in adults with CIndU were presented in a late-breaking oral presentation at the European Academy of Dermatology and Venereology (EADV) 2025 Congress in Paris, France. These early clinical investigations mark a significant step from conceptual validation towards clinical development, positioning MRGPRX2 as a compelling target for future addition to the CIndU therapeutic arsenal.

## Treatment of special populations

CIndU can be challenging to manage and hard to treat, especially for children, although trigger avoidance was the main recommend approaches in the past, current treatment methods focus on a complete protection from trigger-induced whealing or itching. Second-generation H1-antihistamines offer a superior safety profile and are thus recommended as first-line treatment, despite not being licensed for infants under six months in several countries. The choice of a specific second-generation agent should be guided by the child’s age, the availability of age-appropriate formulations (e.g., syrup or fast-dissolving tablets), and national licensing regulations. According to the guideline for urticaria in 2026 (99). Second-generation H1-antihistamines that have demonstrated efficacy and safety in pediatric patients include bilastine, cetirizine, desloratadine, fexofenadine, levocetirizine, loratadine, and rupatadine. Currently, omalizumab is the only biologic approved for antihistamine-refractory CSU in patients aged 12 years and older, while dupilumab is being evaluated in clinical trials for children aged ≥2 years with uncontrolled CSU (133). But no biologics have been approved for pediatric CIndU to date, current management is therefore guided by empirical experience extrapolated from CSU.

CIndU exhibits a marked female predominance, with a female: male ratio of 2:1 to 3:1. and survey data indicate that approximately 30% of female CU patients can expect their disease to worsen during pregnancy, particularly in the first and third trimesters (134). It’s important to provide appropriate guidance on medication use for pregnant and lactating women. With the exception of trigger avoidance, any use of systemic medications is generally recommended to be avoided in pregnant women. Evidence for the use of antihistamines in pregnancy is limited, with only small-sample studies available for cetirizine and a single large meta-analysis for loratadine (135, 136). According to current urticaria guidelines (99), loratadine is the preferred during pregnancy due to safety considerations, with desloratadine, cetirizine, and levocetirizine considered suitable alternatives. It is noted that all H1-antihistamines are secreted into breast milk, but only in low concentrations. Omalizumab has demonstrated a favorable safety profile in pregnancy, with no evidence of teratogenicity reported to date (137, 138). Accordingly, it has been assigned pregnancy category B by both the U.S. Food and Drug Administration (FDA) and the China Food and Drug Administration (CFDA). Based on this, omalizumab may be considered for use during pregnancy and lactation when clinically indicated in China (6). Overall, all subsequent therapeutic decisions should be individualized, prioritizing medications with a favorable risk-benefit profile for both pregnant women and neonates in terms of teratogenicity and embryotoxicity.

## Insight from terminated clinical trails

The developmental trajectory of novel CIndU therapeutics is fraught with challenges, as evidenced by several high-profile agents that failed to translate promising early data into confirmed clinical benefit. Lirentelimab, a monoclonal antibody targeting Siglec-8, demonstrated improved disease control in an open-label Phase IIa study of antihistamine-refractory CSU and CIndU patients, with a notable 92% complete response rate in omalizumab-naïve individuals (139). However, its subsequent randomized, double-blind Phase III trial (NCT05024058) failed to meet the primary endpoint, leading to a halt in its development (140). Similarly, benralizumab, an anti-IL-5Rα antibody, failed to demonstrate a clear treatment advantage in a Phase IIb placebo-controlled study in CU, which included CIndU patients, resulting in the termination of its development program (**Supplementary Table 1**). Furthermore, izuforant (LEO 152020), a selective oral H4R antagonist evaluated in a randomized, placebo-controlled Phase IIa trial for CholU, did not meet its primary or most secondary endpoints, despite a favorable safety profile (141).

These failures highlight the need for novel targets with promising early results to pass rigorous large-scale, placebo-controlled trials. They also suggest that the effectiveness of these therapies might be limited to a subset of the CIndU population. Future success depends on identifying and validating predictive biomarkers to guide patient selection, shifting clinical trials from a “one-size-fits-all” model to a precision medicine approach.

## Approaching to precision medicine

The complexity of CIndU necessitates moving past traditional management models. Precision medicine demands a deep understanding of disease mechanisms and effective tools for measuring disease activity, patient burden, and predicting treatment responses. This section will cover recent advancements in assessment tools and biomarkers for predicting treatment outcomes, highlighting their role in customizing individual treatments in clinical practice.

## Development and optimization of assessment tools for CIndU

Effective management of CIndU depends on tools and biomarkers that accurately measure disease activity and treatment response. Assessment strategies have progressed from using generic scales like UAS7 and UCT to an integrated system combining objective tests with subtype-specific Patient-Reported Outcomes Measures (PROMs) (142).

Notably, CIndU disease-specific PROMs have been recently developed although some of them still need more validation and application (37). For example, altunergil et al. (36) developed the Severity Index for Cold Urticaria (SICU), a comprehensive and reliable index covering triggers, symptoms, quality of life, and treatment efficacy. Grekowitz et al.’s study (35) validated the Cold Urticaria Activity Score (ColdUAS) (143) as a key measure of disease activity, recommending a 2-week evaluation period. Similar tools for CholU and SD, such as CholUAS7 (144), SDAS, and quality of life questionnaires like CholU-QoL (34), ColdU-QoL, and SD-QoL (145, 146), have also been developed (12, 147), providing strong endpoints for assessing CIndU treatments.

The introduction of digital tools like the CRUSE app has further enhanced the integration of PROMs (142), improving assessment precision. The advent of AI, especially Convolutional Neural Networks (CNNs), has transformed healthcare. A recent study (148) introduces the automatic UAS (AUAS) using CNNs to automate hive counting, enhancing objectivity and speed in UAS scoring, and its application could streamline processes and improve health outcomes measurement.

These new PROMs and digital tools developments, integrated with provocation threshold tests, form the basis of the “Assess-Act-Adjust” precision management cycle, enhancing individualized treatment decisions and endpoint selection in clinical trials. Their use should be prioritized in future clinical research.

Despite the development of subtype-specific patient-reported outcome measures (PROMs) for CIndU, their integration into routine clinical practice and research remains challenging (37). In clinical settings, PROMs are substantially underutilized: a global survey of 2, 534 physicians across 73 countries revealed that only 15% routinely employ them for chronic urticaria, including CIndU, with key barriers including time constraints, perceived patient reluctance, and insufficient training in instrument selection (149). Digital platforms such as the CRUSE application offer promising avenues for real-time data collection and integration, with demonstrated validity and reliability (104, 150). However, real-world data indicate that while initial adherence can reach 81%, it tends to decline over prolonged treatment periods (151), and the digital divide may limit accessibility among certain populations. Methodologically, most newly developed subtype-specific PROMs lack established minimally important differences (MID)—the smallest score change perceived as meaningful by patients—which hampers their interpretability as clinical trial endpoints (37, 152). Furthermore, the optimal assessment duration may vary across CIndU subtypes: for example, a 2−week evaluation period is recommended for ColdUAS based on recent validation studies (35), but similar validation is needed for other tools such as CholUAS and SDAS. Realizing the potential of PROMs to advance precision medicine in CIndU will require overcoming these barriers through physician education, user-friendly digital design, rigorous MID validation, and integration with objective provocation tests (104).

## Exploration of biomarkers for predicting treatment response

The significant heterogeneity in treatment response across CIndU subtypes underscores the critical need for biomarkers to guide therapeutic decision-making. However, compared to CSU, research into biomarkers specifically for CIndU remains in its infancy, and current understanding largely relies on extrapolations from broader CU populations.

In predicting response to sgAHs, evidence directly from CIndU studies is sparse. Notably, one of the more specific findings in CIndU is that refractory patients exhibit higher rates of elevated anti-thyroid peroxidase (TPO) antibodies and lower baseline Urticaria Control Test (UCT) scores compared to responders (56). Recently, a prognostic calculator developed for CU patients incorporates clinical variables such as age, angioedema, anxiety/depression, disease duration, NSAID hypersensitivity, and the UAS7 to estimate the likelihood of treatment success (153). High UAS7 scores, elevated C-reactive protein (CRP), and D-dimer levels have been indicated as robust predictors of poor response to sgAHs in patients with CSU (154–156). Moreover, risk factors for antihistamine refractoriness also include a baseline UCT score ≤ 4, emergency referrals, and a family history of CSU (56).

The most promising biomarkers for predicting response to omalizumab, though primarily studied in CSU, revolve around baseline serum total IgE levels (52) and basophil surface FcϵRI expression (84, 157), with higher levels generally associated with better outcomes. Notably, studies suggest that CIndU patients with higher levels of specific IgE, or low basophil counts, may respond better to omalizumab (157). A recent single-centre retrospective study revealed that CU patients who responded to omalizumab had higher pre-treatment eosinophil and basophil counts, while non-responders had elevated baseline anti-thyroglobulin antibodies (158). Additionally, a study demonstrated that CSU patients with a positive basophil activation test (BAT) tend to respond slowly and poorly to omalizumab (6). Furthermore, early dynamic changes in biomarkers may be informative. Lower total IgE levels at 4 weeks (w4 IgE) and a lower w4 IgE/baseline IgE ratio are significantly correlated with non-response to omalizumab in patients with CSU (159, 160). Crucially, predictive biomarker profiles can vary significantly between therapies with different mechanisms. For example, low baseline total IgE and normal D-dimer levels indicate a good response to cyclosporine, unlike omalizumab, aiding clinical decisions.

Although some biomarkers are promising, most are not yet ready for routine clinical application, and their validation specifically in CIndU subtypes is urgently needed. Future research integrating clinical features with molecular biomarkers through prospective studies in CIndU is essential to pave the way for true precision medicine in this complex disease.

## Conclusion

CIndU encompasses a spectrum of MC-mediated disorders characterized by triggered wheals and/or angioedema. Its clinical management remains challenging due to substantial subtype heterogeneity and variable treatment responses. While sgAHs form the first-line therapy, their efficacy is often limited. Omalizumab provides benefit in certain subtypes but remains off-label for isolated CIndU.

The therapeutic landscape is now evolving with novel targeted agents. Anti-KIT biologics demonstrate remarkable efficacy through MC depletion, even in omalizumab-refractory cases. BTK inhibitors offer promise by blocking critical signaling pathways downstream of FcϵRI. However, the failure of several late-phase trials underscores the complexity of CIndU pathology and the inadequacy of non-stratified treatment approaches.

Moving forward, advancing precision medicine in CIndU requires focused efforts on three critical fronts: deepening our understanding of subtype-specific pathogenesis, developing and validating predictive biomarkers to guide treatment selection, and implementing clinical trial designs that incorporate patient stratification and subtype-specific outcome measures. Through these concerted efforts, the field can progress toward matching the right therapy to the right patient, ultimately transforming CIndU management.
