# Supplementary material for: Advancements and challenges in the management of chronic inducible urticaria
Source: Front Immunol. 2026 Apr 24;17:1747542. doi: 10.3389/fimmu.2026.1747542 (PMC13153072; doi:10.3389/fimmu.2026.1747542)
Supplement: Supplementary file 1 [file Table1.docx]

Supplementary Table1. Terminated Clinical Trials of CIndU

| NCT Number | Status | Phase | Population / Cohort | Intervention | Primary Endpoint | Enrollment (Actual) | Study Completion (Actual) | Target / Mechanism | Target Class |
| --- | --- | --- | --- | --- | --- | --- | --- | --- | --- |
| NCT05024058 | Terminated | 3 | Pts with SD, ColdU, or CholU | ligelizumab vs PBO | % pts with UAS7=0 at Wk12 | 39 | Aug 2022 | anti-IgE mAb | mAb |
| NCT04612725 | Terminated | 2b | Pts with CSU (± CIndU) | benralizumab vs PBO | Composite response at Wk24 | 159 | Mar 2023 | IL-5Rα inhibitor | mAb |
